# CHRONO and DEC1/DEC2 compensate for lack of CRY1/CRY2 in expression of coherent circadian rhythm but not in generation of circadian oscillation in the neonatal mouse SCN

**DOI:** 10.1038/s41598-021-98532-5

**Published:** 2021-09-28

**Authors:** Daisuke Ono, Ken-ichi Honma, Christoph Schmal, Toru Takumi, Takeshi Kawamoto, Katsumi Fujimoto, Yukio Kato, Sato Honma

**Affiliations:** 1 Department of Neuroscience II, Research Institute of Environmental Medicine, Nagoya University, Furo-cho, Chikusa-ku, Nagoya, 464-8601 Japan; 2 Department of Neural Regulation, Nagoya University Graduate School of Medicine, Nagoya, 466-8550 Japan; 3 Research and Education Center for Brain Science, Hokkaido University Graduate School of Medicine, Sapporo, 060-8638 Japan; 4 Institute for Theoretical Biology, Humboldt-Universität zu Berlin, Berlin, Germany; 5 RIKEN Brain Science Institute, 2-1 Hirosawa, Wako, Saitama 351-0198 Japan; 6 Department of Physiology and Cell Biology, Kobe University School of Medicine, Kobe, 650-0017 Japan; 7 Department of Dental and Medical Biochemistry, Institute of Biomedical and Health Sciences, Hiroshima University, Hiroshima, 734-8553 Japan

**Keywords:** Cellular neuroscience, Circadian rhythms and sleep

## Abstract

Clock genes *Cry1* and *Cry2*, inhibitory components of core molecular feedback loop, are regarded as critical molecules for the circadian rhythm generation in mammals. A double knockout of *Cry1* and *Cry2* abolishes the circadian behavioral rhythm in adult mice under constant darkness. However, robust circadian rhythms in PER2::LUC expression are detected in the cultured suprachiasmatic nucleus (SCN) of *Cry1*/*Cry2* deficient neonatal mice and restored in adult SCN by co-culture with wild-type neonatal SCN. These findings led us to postulate the compensatory molecule(s) for *Cry1/Cry2* deficiency in circadian rhythm generation. We examined the roles of *Chrono* and *Dec1/Dec2* proteins, the suppressors of *Per(s)* transcription similar to CRY(s). Unexpectedly, knockout of *Chrono* or *Dec1*/*Dec2* in the *Cry1*/*Cry2* deficient mice did not abolish but decoupled the coherent circadian rhythm into three different periodicities or significantly shortened the circadian period in neonatal SCN. DNA microarray analysis for the SCN of *Cry1*/*Cry2* deficient mice revealed substantial increases in *Per*(s), *Chrono* and *Dec*(s) expression, indicating disinhibition of the transactivation by BMAL1/CLOCK. Here, we conclude that *Chrono* and *Dec1*/*Dec2* do not compensate for absence of CRY1/CRY2 in the circadian rhythm generation but contribute to the coherent circadian rhythm expression in the neonatal mouse SCN most likely through integration of cellular circadian rhythms.

## Introduction

The circadian rhythm in mammals is regarded as generated by the core molecular auto-feedback loop involving the clock genes, *Period* (*Per1*, *Per2*), *Cryptochrome* (*Cry1*, *Cry2*), *Bmal1*, and *Clock*, and their protein products^1^. In this loop, the heterodimer of BMAL1/CLOCK binds to E-box enhancer elements located on the *Per1/Per2* or *Cry1/Cry2* promoter and activates transcription of *Per(s)* and *Cry(s)*. Increased *Per1/Per2* and *Cry1/Cry2* proteins in turn suppress the transactivation by BMAL1/CLOCK through direct interactions with them^2,3^. The core molecular loop is interlocked by at least two additional molecular feedback loops, one is the Bmal1 loop which regulates *Bmal1* expression^4,5^ and the other is the Dec loop which suppresses the transcription of *Per(s)* and *Cry(s)* through E-box enhancers^6^.

Double knockout of *Cry1* and *Cry2* abolishes the behavioral circadian rhythm in adult mice, which occurs immediately on release into constant darkness (DD)^7^. However, the circadian rhythm in PER2::LUC expression is still detectable in the cultured suprachiasmatic nucleus (SCN) of *Cry1/Cry2* deficient neonatal mice, which gradually damps around the period of weaning^8,9^. In addition, by exposing to continuous light throughout the neonatal and adolescent period, the circadian rhythms appear in behavior under DD and PER2::LUC expression in cultured SCN of *Cry1/Cry2* deficient mice^10^. Furthermore, the SCN of *Cry1/Cry2* deficient adult mice restores the circadian PER2::LUC expression rhythm by co-culture with the SCN of wild-type (WT) neonatal mice^9^. These findings lead us to postulate the existence of compensatory molecule(s) for a lack of CRY1/CRY2 in the coherent expression of circadian rhythm during the early postnatal period^11,12^.

CHRONO binds to E-box and suppresses transcription of *Per1* and *Per2*. *Chrono* deficient mice showed robust circadian behavioral rhythms with a slightly but significantly lengthened period^13,14^. DEC1 and DEC2 are basic helix–loop–helix transcription factors, which have been suggested as additional negative components of the core molecular loop^6^. They are able to bind to the E-box cis-elements and repress CLOCK/BMAL1-mediated transactivation of *Per1* and *Per2*. The expression of *Dec1* and *Dec2* is negatively regulated by PER1 and PER2, forming a negative feedback loop interlocked with the core molecular loop^15,16^. Thus, CHRONO and DEC1/DEC2 show similar functions in the core molecular loop to CRY1/CRY2. However, circadian oscillation continues in *Dec1/Dec2* deficient mice with a slight change in circadian period similar to *Chrono* deficient mice^17^. Previously, we proposed that CHRONO and DEC1/DEC2 are candidate molecules for the compensation of CRY1/CRY2 function in the core molecular loop^11^.

Beside the cellular oscillation, the SCN neural networks are critical for the expression of circadian PER2::LUC rhythm in the adult as well as the neonatal SCN in culture. Additional knock-out of the VIP receptor gene, *Vipr2*, to the *Cry1/Cry2* deficient mice abolished the coherent circadian PER2::LUC rhythm in the neonatal SCN and CCD camera based imaging exhibited three clusters of cellar oscillations with different periodicities^18^. The features were essentially the same as those exhibited in the adult SCN of *Cry1/Cry2* deficient mice, in which the circadian PER2::LUC rhythm was abolished at the SCN tissue level, whereas the circadian rhythm persisted at the cellular levels with three clusters of different periods. These findings indicate that the expression of circadian rhythm in the SCN of *Cry1/Cry2* deficient mice is regulated also by the SCN neural networks.

In the present study, we examined *Chrono* and *Dec1/Dec2* for their roles in the coherent expression of circadian rhythm in the neonatal SCN of *Cry1/Cry2* deficient mice. We found that *Chrono* and *Dec1/Dec2* expression were substantially increased in the SCN of *Cry1/Cry2* deficient mice, indicating disinhibition of the transactivation by BMAL1/CLOCK. Unexpectedly, additional knockout of *Chrono* or *Dec1/Dec2* in the *Cry1/Cry2* deficient mice (triple-KO of *Cry1/Cry2/Chrono* or quadruple-KO of *Cry1/Cry2/Dec1/Dec2/*) did not abolish the circadian PER2::LUC rhythm but modified expression of coherent rhythms in the SCN. The triple-KO uncoupled the coherent circadian rhythm into three components of different periodicities and the quadruple-KO extremely shortened the circadian period. These findings indicate that *Chrono* and *Dec1/Dec2* do not compensate for absence of *Cry1/Cry2* in the circadian rhythm generation but contribute to the coherent circadian rhythm expression in the neonatal SCN most likely through the coupling of cellular circadian rhythms.

## Results

### Microarray analysis in the *Cry1/Cry2* deficient SCN

**Figure 1:**
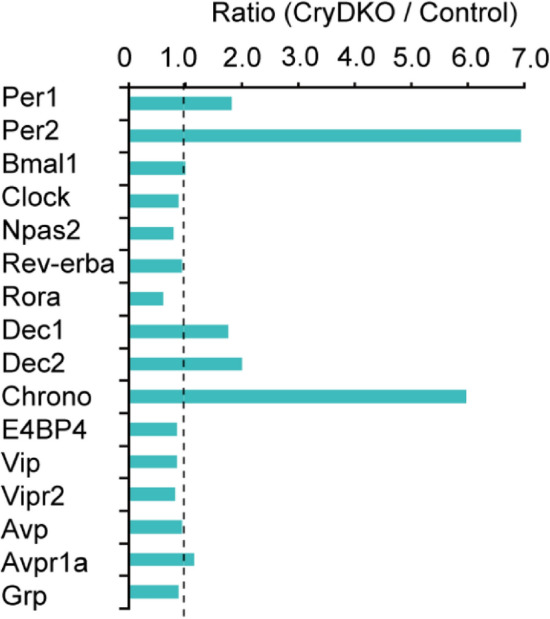
Expression of representative genes in neonatal *Cry1/Cry2* deficient and control SCN. Relative gene expression in *Cry1/Cry2* deficient mice to that in the control is illustrated as a bar graph. Gene expression in *Cry1/Cry2*-deficient SCN was divided by that in the control SCN (Ratio). Clock genes, several clock related genes, SCN related major neuroendocrine genes, and their receptor genes are demonstrated in the graph.

To determine how gene expression in the SCN of *Cry1/2* deficient mice is altered by the loss of *Cry1/2*, transcription was measured in the SCN. SCNs were collected from neonatal (postnatal day 7: P7) and adult (2–4 month old) mice at Zeitgeber Time (ZT) 6, where the time of light-on in the light–dark cycle defines ZT 0. DNA microarray analysis revealed that the expression ratio (*Cry1/Cry2* deficient vs control) increased significantly in *Dec1* (ca. 180%), *Dec2* (ca. 200%) and *Chrono* (ca.600%) in addition to *Per1* (ca. 190%) and *Per2* (ca. 700%) in the neonatal SCN (Fig. 1). Among 27,296 genes in the DNA microarray chip, 653 genes were upregulated (log2(FC) > 0.5) and 696 genes were downregulated (log2(FC) < 0.5) in *Cry1/Cry2* deficient SCN. Interestingly, the expressions were not enhanced in *Bmal1*, *Rev-erbα* and *Avp* which are transcribed though E-box elements, indicating that gene expression through E-box is not always dependent on CRY1 and CRY2. Expression levels of other clock genes and clock-related genes including those involved in phosphorylation of clock gene products were not different between the control and *Cry1/Cry2* deficient SCN. Similar results were obtained in the adult SCN of *Cry1/Cry2* deficient mice (Supplemental Fig. 1).

### Circadian PER2::LUC rhythms in *Cry/Chrono* triple-KO and *Cry/Dec* quadruple-KO mice

To test the roles of *Chrono* and *Dec1/Dec2* in the expression of circadian rhythms in the *Cry1/Cry2* deficient neonatal mice, we made *Cry1/Cry2/Chrono* (*Cry/Chrono*) triple-KO mice and *Cry1/Cry2/Dec1/Dec2* (*Cry/Dec*) quadruple-KO mice, both carrying a PER2::LUC reporter. We measured PER2::LUC bioluminescence in the neonatal SCN slices in culture using a photomultiplier tube^9,18^ and analyzed circadian rhythms by *Chi*-square periodogram. The periods shown are the results of *Chi*-square periodogram unless otherwise stated.

**Figure 2:**
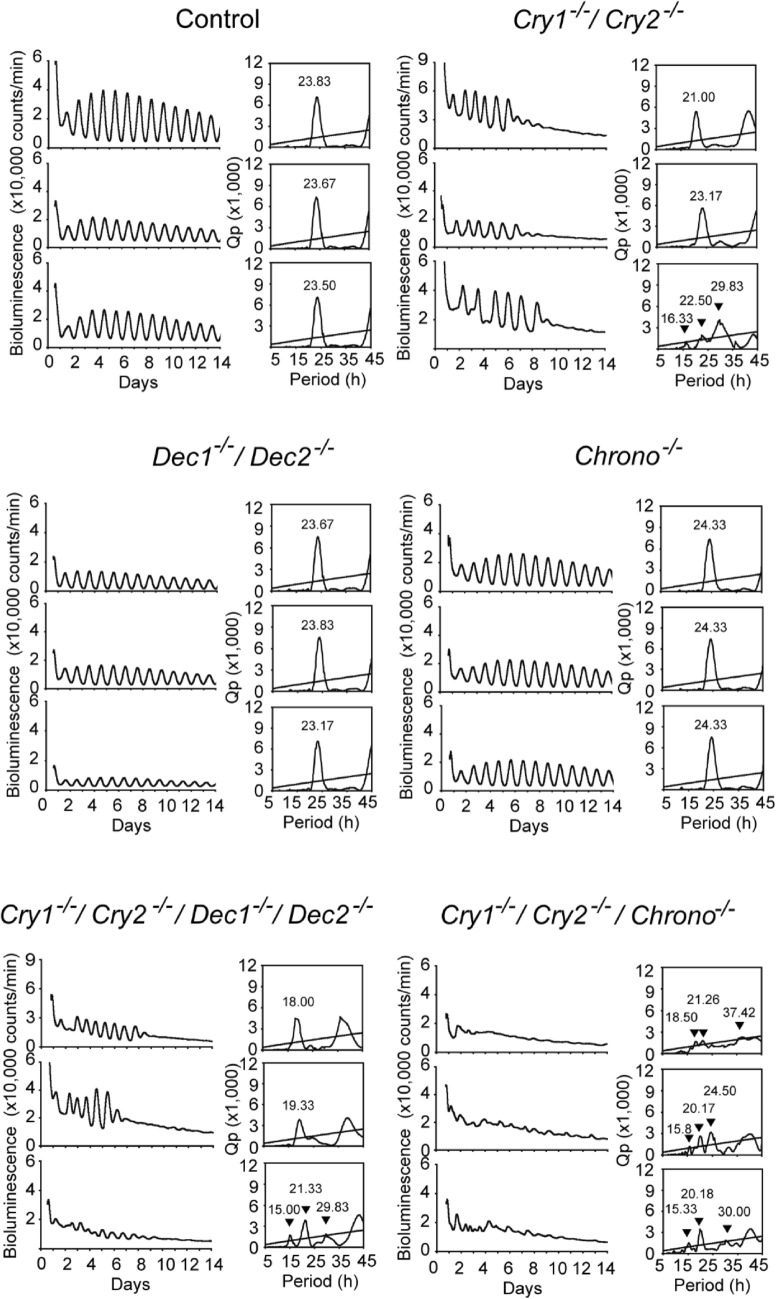
PER2::LUC rhythms of the neonatal SCN slice culture in different genotypes. PER2::LUC rhythms in the neonatal SCN of different genotypes. Three representative rhythms are shown. Genotypes are indicated in each panel. Chi square periodograms for each rhythm in the panel of the left column. The oblique line in the peridogram indicates a significant level of *P* = 0.01. Triangles and numbers are the peak period of each slice.

Robust circadian PER2::LUC rhythms were detected in the neonatal SCN of the control, *Cry1/Cry2* deficient, and *Dec1/Dec2* deficient mice (Fig. 2), as reported previously^10,19^. The circadian periods as well as phase in individual *Cry1/Cry2* deficient SCN were substantially varied in marked contrast with those in the control and *Dec1/Dec2* deficient SCN (Fig. 2). The large phase variation is partly due to different circadian periods of individual SCNs and/or possibly due to a failure of entrainment to a LD cycle and/or to the effects from nursing mother before the time of SCN preparation^9^. Significant circadian PER2::LUC rhythms were also detected in the neonatal SCN of *Chrono* deficient mice and *Cry/Dec* quadruple-KO mice (Fig. 2). The circadian PER2::LUC rhythms in the *Chrono* deficient SCN were similar to that in the control except for slightly but significantly longer period (24.3 ± 0.1 h vs. 23.7 ± 0.2 h; P < 0.01), whereas the circadian rhythms in *Cry/Dec* quadruple-KO SCN showed substantial variability in the period as in the case of *Cry1/Cry2* deficient SCN (Fig. 2). In contrast to the above five genotypes, *Cry/Chrono* triple-KO SCN showed circadian PER2::LUC rhythms of relatively low amplitude. Periodogram analysis revealed two other rhythmic components with shorter and longer periods than that near 24 h (Fig. 2). Circadian PER2::LUC rhythms in other SCN were illustrated in Supplemental Fig. 2. To confirm the separate periodicities, the peaks that were larger than the mean level of periodicity (above the zero line of the detrended time series data) were selected and peak-to-peak intervals were calculated. Three-dimensional distribution maps of the intervals indicate the intra- as well as inter-individual differences of rhythmicity (Supplemental Fig. 3b). The intervals were concentrated at around 24 h in control, *Chrono* KO and *Dec1/Dec2* DKO mice, whereas they distributed in a wider range in the *Cry1/Cry2/Chrono* triple-KO, *Cry(s)/Dec(s)* quadruple-KO and *Cry1/Cry2* KO mice (Supplemental Fig. 3).

### Circadian properties in *Cry/Chrono* triple-KO mice and* Cry/Dec* quadruple-KO mice

**Figure 3:**
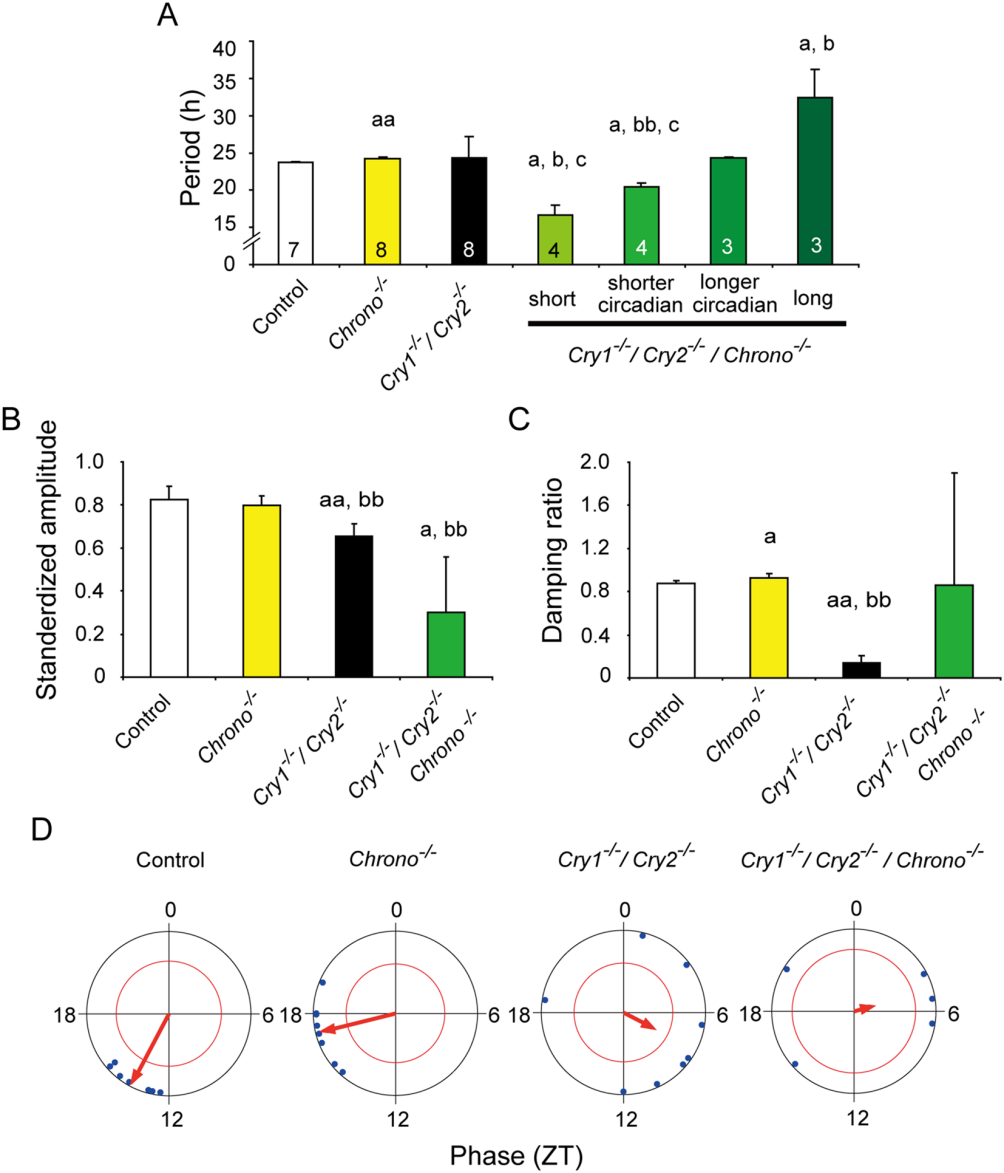
Rhythm properties of neonatal SCN in culture from the control, *Chrono* KO, *Cry1/Cry2* deficient, and *Cry1/Cry2*/*Chrono* triple-KO mice. (**A**) Mean periods of PER2::LUC rhythms with standard deviations (SD) calculated by periodogram are shown as columns and vertical lines. A number in each column indicates number of SCN examined. (**B**) Mean standardized amplitude of PER2::LUC rhythms are expressed in a bar graph (mean ± SD). (**C**) Damping ratio of PER2::LUC rhythms is also shown in a bar graph (mean ± SD). Statistical comparisons, a; *P* < 0.05, aa; P < 0.01 vs. control, b; *P* < 0.05, bb; *P* < 0.01 vs.*Chrono* deficient mice, c; *P* < 0.05, cc; *P* < 0.01 vs. *Cry1/Cry2* deficient mice (Kruskal–Wallis with a post-hoc Steel-test). (**D**) Peak phase of PER2::LUC rhythms in the 1^st^ day of culture is illustrated as Rayleigh plot. Blue dots indicate the peak times of individual rhythms. Red arrow in each circle indicates the mean circadian phase. A red circle is 95% confidence level. If an arrow extends over the red circle, the distribution of peak phases is regarded as significantly consolidated.

**Figure 4:**
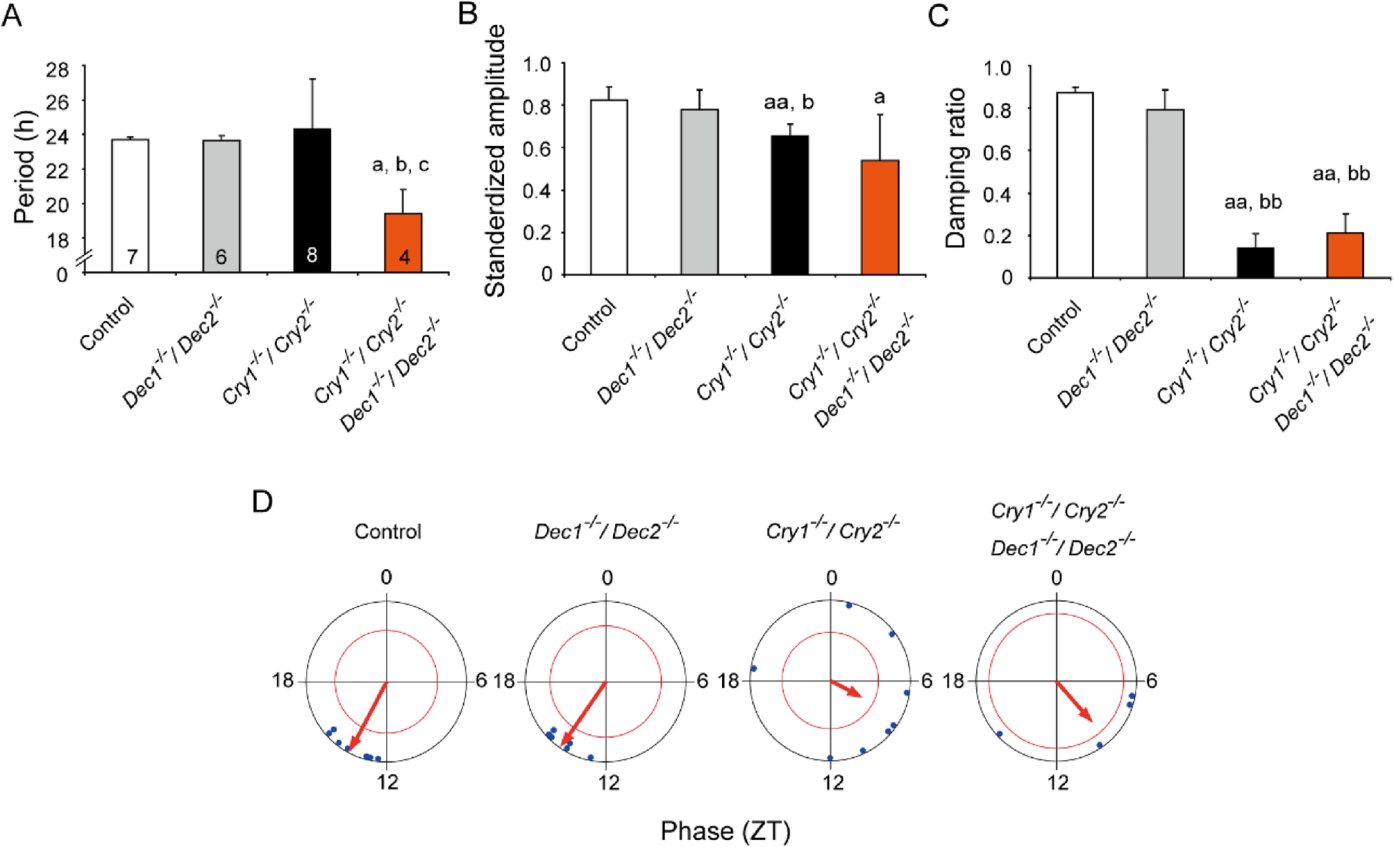
Rhythm properties of neonatal SCN in culture from the control, *Dec1/Dec2* deficient, *Cry1/Cry2* deficient, and *Cry1/Cry2/ Dec1/Dec2* quadruple-KO mice. (**A**) Mean periods of PER2::LUC rhythms with standard deviations (SD) calculated by periodogram are shown as columns and vertical lines. A number in each column indicates the number of SCN examined. (**B**) Standardized amplitude of PER2::LUC rhythms are expressed in bar graph (mean ± SD). (**C**) Damping ratio of PER2::LUC rhythms is also shown in a bar graph (mean ± SD). Statistical comparisons, a; *P* < 0.05, aa; *P* < 0.01 vs. control, b; *P* < 0.05, bb; *P* < 0.01 vs. *Dec1/Dec2* deficient mice, c; *P* < 0.05 vs. *Cry1/Cry2* deficient mice (one-way ANOVA with a post hock Tukey–Kramer test or Kruskal–Wallis with a post-hoc Steel-test). (**D**) Peak phase of PER2::LUC rhythms on the 1st day of culture in ZT is illustrated as Rayleigh plot, where ZT 0 indicates the time of light-on in the light–dark cycles. Blue dots indicate the peak phases of individual rhythms. Red arrow in each circle indicates the mean circadian phase of each genotype. A red circle is 95% confidence level. If an arrow extends over the red circle, the distribution of peak phases is regarded as significantly consolidated. Data of the control and *Cry1/Cry2*-deficient SCN are same as in Fig. 3.

Effects of *Chrono* or *Dec1/Dec2* deletion on the circadian PER2::LUC rhythms of *Cry1/Cry2* deficient SCN were statistically analyzed and summarized in Figs. 3 and 4, respectively. In marked contrast with other genotypes examined, the SCN of the individual *Cry/Chrono* triple-KO mice showed essentially three major periodicities in *Chi*-square periodogram, but since in some animals the two distinct periodicities were detected in the near-circadian range, they were separately analyzed [short, 16.6 ± 1.4 h (n = 4); shorter circadian, 20.4 ± 0.6 h (n = 4); longer circadian, 24.3 ± 0.2 h (n = 3); long, 32.5 ± 3.7 h (n = 3)]. Some individuals showed the short, shorter and longer circadian, but others, the shorter, longer circadian and long. By the analysis of peak-to-peak intervals, there detected the corresponding 4 periodicities (short, 15.7 ± 0.8 h; shorter circadian, 20.6 ± 1.0 h; longer circadian, 24.3 ± 0.2 h; long, 31.9 ± 4.9 h) (Figs. 2 and 3A, Supplemental Fig. 2). The variability (SD) of peak-to-peak intervals distributed from 0.9 to 3.7 h in each slice and the mean variability was 2.0 h (SD = 0.9 h). The short, shorter circadian and long period were significantly different from the periods of control (23.7 ± 0.2 h) and *Chrono* deficient mice (24.3 ± 0.1 h). These periods were also significantly different from those of *Cry1/Cry2* deficient mice (24.3 ± 2.9 h) except for the long period (Kruskal–Wallis test with post-hoc Steel-test; *P* < 0.01 or 0.05). The standardized amplitude was significantly lower in *Cry/Chrono* triple-KO mice (0.30 ± 0.26) than in the control mice (0.82 ± 0.06; *P* < 0.05) and *Chrono* deficient mice (0.80 ± 0.04; *P* < 0.01), but not in *Cry1/Cry2* deficient mice (Fig. 3B). The damping ratio of *Chrono* deficient mice (0.93 ± 0.04) was significantly larger than that of *Cry1/Cry2* deficient mice (0.14 ± 0.07; *P* < 0.01), and even that of the control mice (0.87 ± 0.03; *P* < 0.01) (Fig. 3C, Supplemental Fig. 3), indicating that the amplitude was maintained at a relatively constant level throughout this period in *Chrono* deficient mice. The damping ratio of *Cry/Chrono* triple-KO mice was not different from those of other genotypes due to a large standard deviation (Fig. 3C, Supplemental Fig. 3). The peak phase of PER2::LUC rhythm on the 1st day of culture was slightly but significantly delayed in *Chrono* deficient mice (17.1 ± 1.5 h) as compared to the control mice (13.9 ± 1.2 h; *P* < 0.01, Watson-Williams F-test) (Fig. 3D). The peak phases in *Cry1/Cry2* deficient and *Cry/Chrono* triple-KO mice were variable and did not consolidate as judged from the length of the arrow in the Rayleigh plot (Fig. 3D).

On the other hand, the periods of *Cry/Dec* quadruple-KO mice were 19.4 ± 1.4 h (n = 4) by *Chi*-square periodogram which was significantly shorter than those of the control (23.7 ± 0.2 h, n = 7; *P* < 0.05), *Dec1/Dec2* deficient (23.6 ± 0.3 h, n = 6; *P* < 0.05), and *Cry1/Cry2* deficient mice (24.3 ± 2.9 h, n = 8; *P* < 0.05) (Fig. 4A). The peak-to-peak intervals in *Cry/Dec* quadruple-KO mice (18.8 ± 0.5 h, n = 4) were also significantly shorter than that of the control (23.7 ± 0.1 h, n = 7). The standardized amplitude of PER2::LUC rhythm was significantly lower in both *Cry1/Cry2* deficient mice (0.66 ± 0.06; *P* < 0.01 v.s. control, *P* < 0.05 v.s. *Dec1/Dec2* deficient mice) and *Cry/Dec* quadruple-KO mice (0.54 ± 0.22; *P* < 0.05 v.s. control) as compared with the control mice (0.82 ± 0.06) or *Dec1/Dec2* deficient mice (0.78 ± 0.09) (Fig. 4B). The damping ratio was significantly smaller (damping is larger) in *Cry1/Cry2* deficient (0.14 ± 0.07; *P* < 0.01) and *Cry/Dec* quadruple-KO (0.21 ± 0.09; *P* < 0.01) mice than in the control (0.87 ± 0.03) or *Dec1/Dec2* deficient mice (0.79 ± 0.09) (Fig. 4C, Supplemental Fig. 3). The peak phase of PER2::LUC rhythm on the 1st day of culture was located at around ZT13-14 on average in the control and *Dec1/Dec2* deficient mice. They were significantly consolidated as indicated by an arrow extending over the critical red circle (95% confidence level) in the Rayleigh plot (Fig. 4D). However, the peak phases in individual SCNs were not significantly consolidated in *Cry1/Cry2* deficient and in *Cry/Dec* quadruple-KO mice.

## Discussion

In the present study, using *Cry/Chrono* triple-deficient and *Cry/Dec* quadruple-deficient mice, we demonstrate that *Chrono* and *Dec1/Dec2* play distinct roles in the coherent expression of circadian PER2::LUC rhythm in the neonatal SCN of *Cry1/Cry2* deficient mice. *Chrono* is involved in the integration of cellular circadian rhythms of different periodicities and *Dec1/Dec2* is in the lengthening of circadian period at the earliest postnatal period. These genes compensate the expression of coherent circadian rhythm in the SCN of *Cry1/Cry2* deficient mice most likely through the networks of cellular oscillation.

The expression of *Chrono* and *Per2* were substantially and of *Dec(s)* and *Per1* moderately increased in the SCN of *Cry1/Cry2* deficient mice of both neonates and adults (Fig. 1, Fig. 1S), indicating that a lack of CRY1/CRY2 disinhibits the transcription of these genes and that the core molecular feedback loop unlikely functions in *Cry1/Cry2* deficient mice. The circadian PER2::LUC rhythm in the SCN of *Cry/Chrono* triple-deficient mice was fragmented into three components with different periodicities and showed large cycle-to-cycle variability (Fig. 3). Of great interest, the three periodicities (16.6 ± 1.4 h, 20.4 ± 0.6 h or 24.3 ± 0.2 h, and 32.5 ± 3.7 h) are similar to those detected in the SCN of VIP receptor (*Vpac2*) and *Cry1/Cry2* deficient mice (triple deficient) by CCD camera based imaging, in which three clusters of cellular rhythm with different periodicities (16.9 ± 1.6 h, 21.0 ± 0.4 h, and 28.3 ± 0.4 h) were detected^18^. Knock-out of *Vpac2* abolished the coherent circadian rhythm in the neonatal SCN of *Cry1/Cry2* deficient mice. The similar three clusters are also observed in the adult SCN of *Cry1/Cry2* deficient mice^18^. Importantly, coherent circadian rhythms in PER2::LUC were restored in the adult SCN by co-culture with the WT neonatal SCN, which was accompanied by a single cellular cluster. These findings lead us to postulate that the coherent circadian rhythm in the SCN is built-up by the coupling of these three clusters of cellular oscillation. CHRONO is involved in the coupling of cellular oscillations.

The neuropeptides released from the co-cultured WT SCN are involved in the coupling of cellular oscillations through the networks of recipient SCN. The responsible neuropeptide is AVP, since administration of AVP receptor antagonists abolished the restored circadian PER2::LUC rhythm^18^. The abolishment of circadian rhythms in the adult *Cry1/Cry2* deficient mice is likely due to a lack of the circadian release of AVP in the SCN, since *Cry1/Cry2* deficiency substantially suppresses AVP gene expression^18^. By contrast, the role of VIP is different in the neonatal and adult SCN, which may explain why the coherent circadian PER2::LUC rhythm is preserved in the neonatal SCN but not in the adult of *Cry1/Cry2* deficiency mice. In the neonatal SCN, VIP is released in a circadian fashion, whereas in the adult VIP release lacks an endogenous nature of circadian rhythm and is stimulated by lights^20,21^.

Tetrodotoxin (TTX) treatment to the neonatal SCN of *Cry1/Cry2* deficient mice abolished the coherent circadian PER2::LUC rhythm in the SCN due to desynchronization of cellular circadian rhythms, the features of which were quite similar to those of TTX untreated adult SCN^9^. The periods of cellular oscillation in the TTX treated neonatal SCN distributed in a wide range (from13 up to 37 h), which is similar to the period distribution of dispersed cell culture of CRY(s)-deficient SCN. These findings also support the idea that the SCN neural networks are critical in the expression of coherent circadian rhythm in the neonatal SCN of *Cry1/Cry2* deficient mice^9^. Based on these and above mentioned findings, we proposed a model of circadian organization in the SCN of *Cry1/Cry2* deficient mice^18^. The cellular oscillations in the SCN are diverse in periods without mutual synchronization (basal state). The diversity of period suggests that the oscillation is a kind of molecular noise ^22^. In the presence of functional SCN networks, there appear three clusters of cellular oscillation due to mutual synchronization (cluster state). The oscillations of three cellular clusters are further synchronized in several ways to express the coherent circadian rhythms (coherence state). *Chrono* and *Dec1/Dec2* are differentially involved in synchronization of cell oscillation clusters. CRY1/CRY2 could also play as a synchronizer of cellular oscillation through AVP release^18^.

Recently, a post-translational model was proposed for the circadian oscillation of *Cry1/Cry2* deficient mice^23^. In this study, in addition to the robust circadian rhythms in PER2::LUC, the temperature compensation and period determination by CK1ε/δ activity were demonstrated in the cultured fibroblasts of *Cry1/Cry2* deficient mice. Their findings support our hypothesis that the CRY1/CRY2 are dispensable in the circadian oscillation.

On the other hand, in *Cry/Dec* quadruple-KO mice the PER2::LUC rhythms in the SCN show a substantially shorter period (19.4 ± 1.4 h) than that of *Cry1/Cry2* deficient mice (24.3 ± 2.9 h) (Figs. 2 and 4). In the earliest postnatal period, the SCN of *Cry1/Cry2* deficient mice showed a very short period of ca. 16 h, which was gradually lengthened to ca. 24 h in a week. We postulate that in the earliest postnatal period, the integration of cellular oscillations is brought about, so that the oscillations of the shortest period are dominant in the coherent circadian rhythm, and the dominancy is gradually shifted to the oscillations of longer periods. *Dec1/Dec2* is possibly involved in this gradual change of integration of cellular oscillations in the early neonatal period. A lack of *Dec1/Dec2* may interrupt the cellular coupling in the earliest state of postnatal period. The site of cellular integration could be the neural or humoral networks in the SCN. The molecular core loop is unlikely involved in the change of circadian period, since *Per(s)* expression is disinhibited and the core molecular loop does not work in *Cry1/Cry2* deficient mice. These findings indicate that DEC1 and DEC2 contribute to the lengthening of the circadian period in the earliest postnatal period.

Circadian oscillation could be generated without the core transcription-translation feedback loop (TTFL). *Bmal1* is a positive element of molecular feedback loop and *Bmal1* deficiency resulted in abolishment of circadian behavioral rhythms in DD^24^. However, periodicities in the circadian domain were detected in PER2::LUC expression in the *Bmal1* deficient SCN slice culture^22^. The finding was interpreted in such a way that “quasi-circadian” rhythms in the *Bmal1* deficient SCN have a stochastic nature produced by intercellular coupling and molecular noise. The general features of the *Cry1/Cry2* deficient SCN are similar to those of the *Bmal1* deficient SCN. Recently, *Bmal1* deficient mice demonstrated circadian transcript rhythms in fibroblasts and liver^25^. Circadian rhythm was detected without the nucleus in the red blood cells in mammals, and in acetabularia, a single cell algae^26,27^. A lack of a TTFL of clock genes could be compensated by neural networks and/or other oscillatory mechanisms such as post-translational oscillation^23^ and a single molecule oscillation^28^.

In this study, we did not analyze the integration of cellular oscillations in the SCN. It would be interesting to know how the spatio-temporal patterns of PER2::LUC in the SCN were changed in the *Cry/Chrono* triple-deficient and *Cry/Dec* quadruple-deficient mice. Furthermore, it would be interesting to know whether the stability of PER2 in *Cry/Chrono* triple-deficient and *Cry/Dec* quadruple-deficient SCN is modulated or not, since the stability of PER2 proteins is reported to affect the length of circadian period^29^.

In conclusion, CHRONO and DEC1/DEC2 do not compensate for absence of CRY1/CRY2 in the circadian rhythm generation but contribute to the expression of coherent circadian rhythms in the neonatal mouse SCN. CHRONO is involved in the integration of clusters of cellular oscillations with different periods. On the other hand, DEC1/DEC2 lengthens the period in the earliest neonatal period of *Cry1/Cry2* deficient mice, probably through the integration of cellular rhythms. Despite of the disinhibition of *Per1/Per2* expression, the circadian period of PER2::LUC rhythm in the *Cry1/Cry2* deficient SCN was comparable to that of the control SCN, suggesting the presence of other oscillatory mechanisms than TTFL in *Cry1/Cry2* deficient mice.

## Methods

### Animals

*Cry1/Cry2* KO*, Dec1* KO and *Chrono* KO mice of C57BL/6J background were obtained from Tohoku University^30^, Hiroshima University^31^, and Riken^14^, respectively. *Dec2* KO mice were originally established by replacing the 2.7 kb region in exons 1–5 of the *Dec2* gene including the entire coding region with Neo cassette as described elsewhere^31^. *Chrono* KO mice were made by C57BL/6 background ES cell clone. *Dec1* and *Dec2* KO mice were backcrossed with C57BL/6J mice, and confirmed the existence of more than 99.5% of C57BL/6J background by using a speed congenic method. For the respective genotypes, only the animals were used in which all corresponding genes were knocked out except for *Cry2,* and deficiency of *Cry2* were confirmed by PCR genotyping. The bioluminescence reporter system was introduced to each of the knockout mice by breeding with PER2::LUC homozygote mice carrying a PER2 fusion luciferase reporter^19^. We used only PER2::LUC homozygote mice that could be identified by PCR genotyping. Mice were bred and reared in the animal quarter at Hokkaido University Graduate School of Medicine, where environmental conditions were controlled (lights-on 6:00–18:00 h; light intensity approximately 100 lx at the bottom of cage: humidity 60 ± 10%). Both male and female mice were used for the present study. Experiments were conducted in compliance with the rules and regulations established by the Animal Care and Use Committee of Hokkaido University under the ethical permission of the Animal Research Committee of Hokkaido University (Approval No. 08-0279) with the ARRIVE guidelines.

### Microarray analysis

Brain tissues of 400 μm thick were made with a microslicer (DTK-1000; Dosaka EM) at ZT6 from neonatal (postnatal day 7: P7) or adult (2–4 month old) wild type or *Cry1/Cry2* deficient mice, and only the SCN area was trimmed by a surgical scalpel under a stereo microscope. All animals contained homozygote of PER2::LUC reporter. RNA was extracted using RNeasy micro kit (QIAGEN) from 8–10 mice, then performed microarray analysis (Agilent Technologies).

### SCN slice preparation for culture

For the measurement of PER2::LUC bioluminescence, animals were euthanized to harvest SCNs between 8:00 and 16:00 h. Coronal SCN slices of 300 μm thick were made with a tissue chopper (Mcllwain) from neonatal mice (P7). The SCN tissue was dissected at the mid rostrocaudal region and a paired SCN was cultured on a Millicell-CM culture insert (Millipore Corporation). The culture conditions were the same as those described previously^9,18^. Briefly, the slice was cultured in air at 36.5 °C with 1.2 ml Dulbecco’s modified Eagle’s medium (Invitrogen) supplemented with 10 mM HEPES, 2.74 mM NaHCO_3_, 0.1 mM D-luciferase K, and 5% supplement solution the composition of which is reported elsewhere^32^.

### Measurement of bioluminescence

Bioluminescence of the SCN tissue level was measured using a PMT (Lumicycle; Actimetrics) at 10 min intervals with an exposure time of 1 min. as described previously^10^. The intensity of bioluminescence was expressed in relative light units (RLU; counts/min). The measurement was continued at least for 14 days.

### Data analysis

Periods of circadian PER2::LUC rhythm in the SCN culture was determined with *Chi*-square periodogram using bioluminescence data of initial 7 cycles. Bioluminescence records of the first 12 h were not used for rhythm analyses because of an initial high level of bioluminescence. The raw bioluminescence data were smoothed using a five-point moving average method and then detrended using a 24 h running average subtraction method^9^. The circadian period and its stability were also analyzed by measuring a peak and the following peak. The peaks above the mean level of the rhythmicity in the detrended time series data (the zero-line) were selected and the peak-to-peak intervals were calculated. By this method, some significant peaks in the periodogram were revealed to be harmonics and excluded from the further analyses. The rhythm amplitude was defined as the difference between the peak and trough in a cycle of 4^th^ day in culture and standardized by dividing the amplitude by the peak level as described previously^9^. The damping ratio was defined as the ratio of the circadian amplitude of 14th and 4th day in culture of PER2::LUC rhythms in the SCN, since a reduction of the circadian amplitude of *Cry1/Cry2* deficient SCN were expected around 10th day in culture. The peak phase in the 2nd day of culturing was analyzed as described previously^9^.

### Statistics

A one-way ANOVA with a post-hoc Tukey–Kramer test or Kruskal–Wallis with a post-hoc Steel-test was used to analyze multiple group data (Statview or Statcel 3). A Rayleigh plot was made using the Oriana4 software (Kovach Computing Service).

### Ethics approval and consent to participate

Experiments were conducted in compliance with the rules and regulations established by the Animal Care and Use Committee of Hokkaido University under the ethical permission of the Animal Research Committee of Hokkaido University (Approval No. 08-0279).

## Supplementary Information

Supplementary Information.

## Data Availability

All data generated during this study are included in this published article and its supplementary information files, and all materials generated during this study are available upon request.

## Abbreviations

AVP: Arginine vasopressin
DD: Continuous darkness
KO: Knockout
LD: Light–dark cycle
PER2::LUC: Per2 luciferase
PMT: Photomultiplier tube
SCN: Suprachiasmatic nucleus
TTFL: Transcription-translation feedback loop
VPAC2: VIP receptor 2
VIP: Vasoactive intestinal polypeptide
