# Supplementary material for: CHRONO and DEC1/DEC2 compensate for lack of CRY1/CRY2 in expression of coherent circadian rhythm but not in generation of circadian oscillation in the neonatal mouse SCN
Source: Sci Rep. 2021 Sep 28;11:19240. doi: 10.1038/s41598-021-98532-5 (PMC8479135; doi:10.1038/s41598-021-98532-5)
Supplement: Supplementary file 1 — Supplementary Information. [file 41598_2021_98532_MOESM1_ESM.pdf]

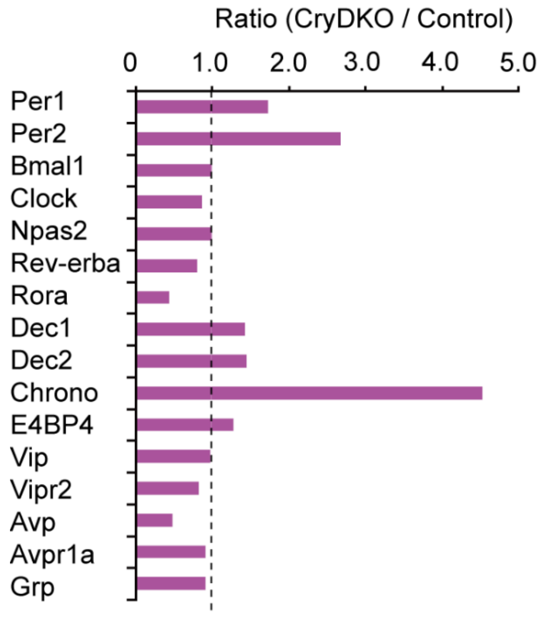

Supplemental figure 1: Expression of representative genes in adult *Cry1/Cry2* KO and control SCN

Relative gene expression in *Cry1/Cry2* deficient mice to that in the control is illustrated as a bar graph. Gene expression in *Cry1/Cry2*-deficient SCN was divided by that in the control SCN (Ratio). Clock genes, some clock related genes, SCN related major neuroendocrine genes, and their receptor genes are demonstrated in the graph.

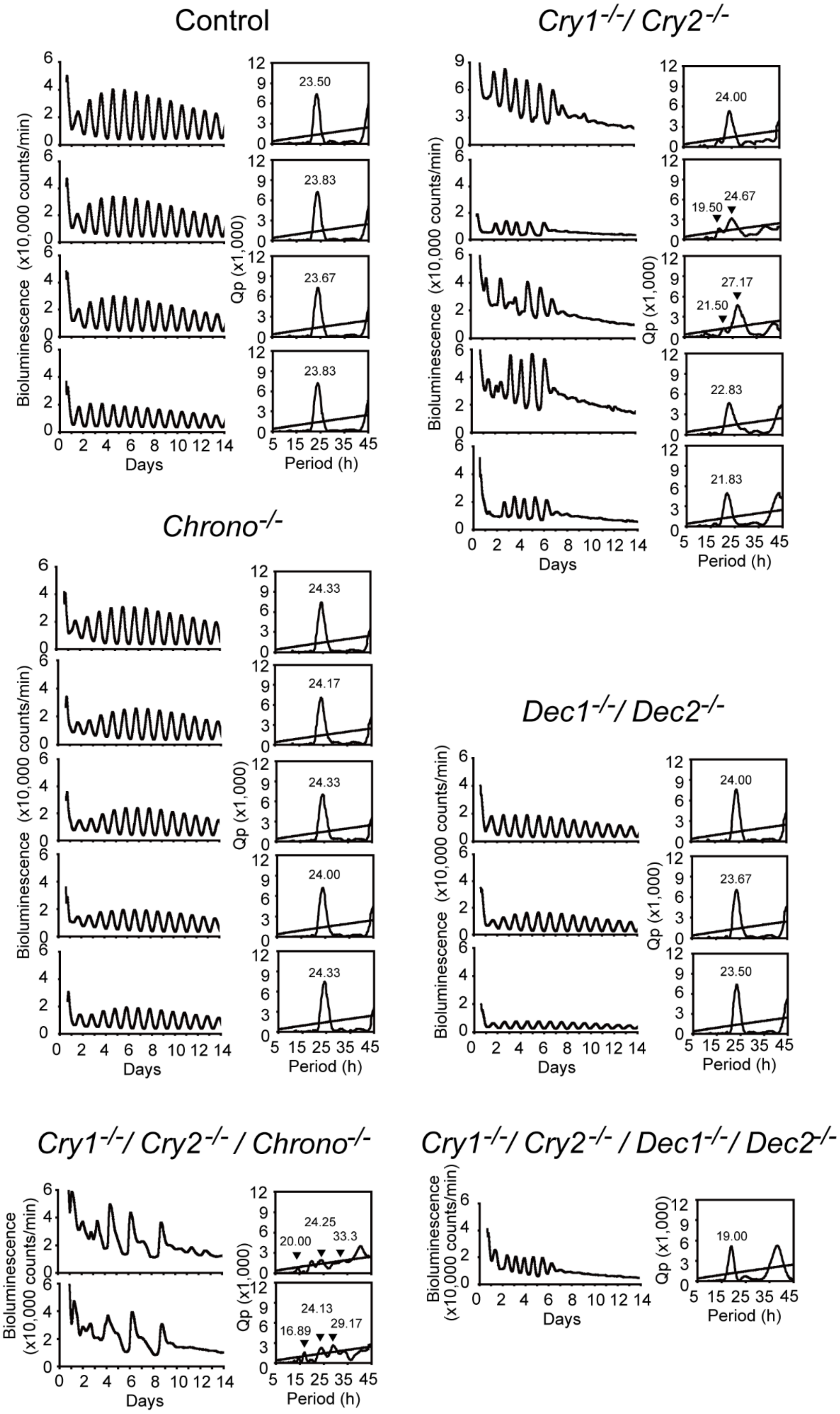
Control
Bioluminescence (x10,000 counts/min)
Qp (x1,000)
23.50
23.83
23.67
23.83
Days
Period (h)
Cry1-/-/ Cry2-/-
24.00
19.50
24.67
21.50
27.17
22.83
21.83
Chrono-/-
24.33
24.17
24.33
24.00
24.33
Dec1-/-/ Dec2-/-
24.00
23.67
23.50
Cry1-/-/ Cry2-/- / Chrono-/-
20.00
24.25
33.3
16.89
24.13
29.17
Cry1-/-/ Cry2-/- / Dec1-/-/ Dec2-/-
19.00

Supplemental figure 2: Other examples of PER2::LUC rhythms in the neonatal SCN slices of different genotypes

PER2::LUC rhythms of neonatal SCN slice (left) togather with periodogram (right) are exhibited in each genotype. A number in each panel of periodogram indicates dominant periodicity. The oblique line in the peridogram indicates a significant level of P = 0.01.

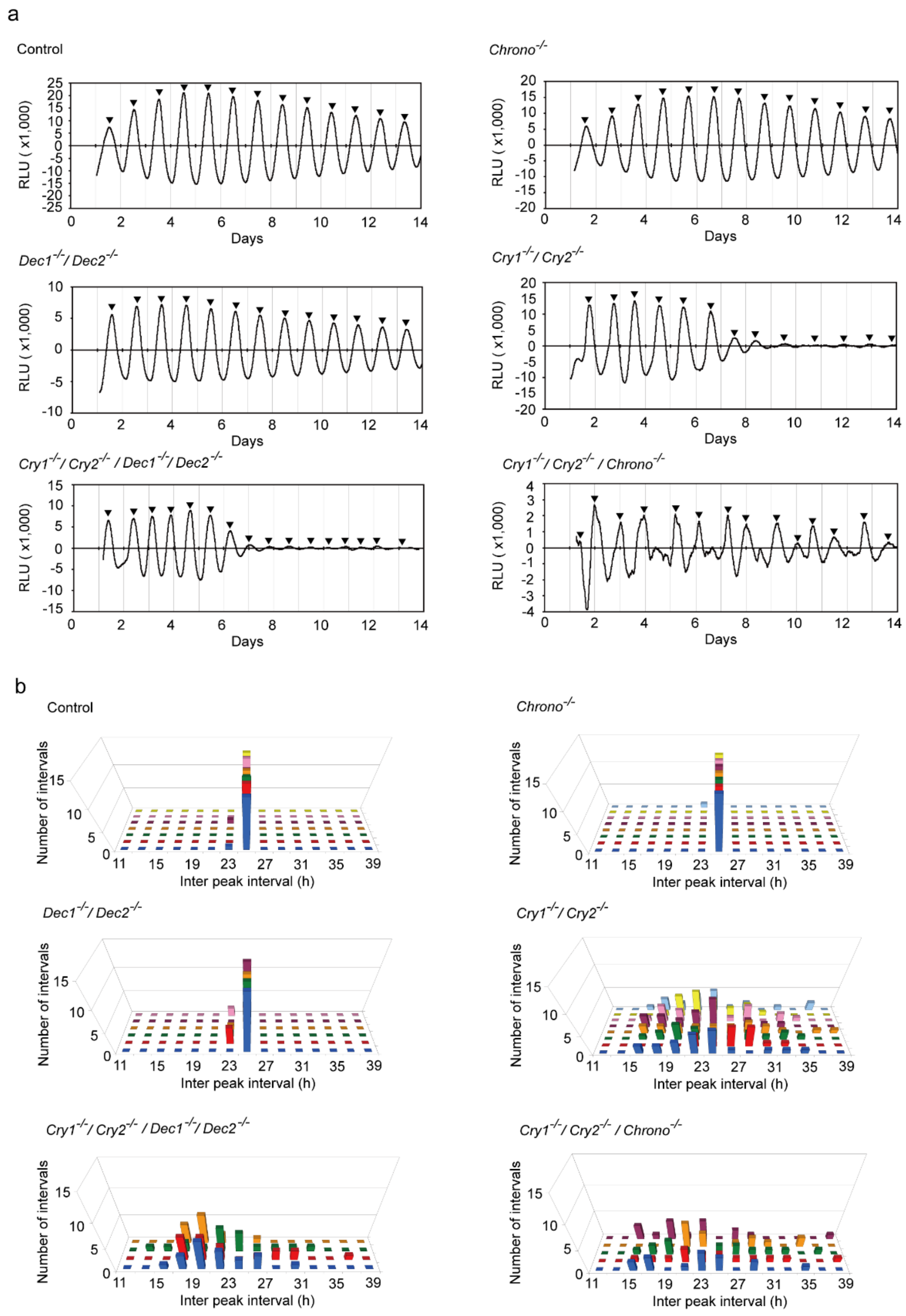

Supplemental figure 3: Representative examples of detrended PER2::LUC rhythms and 3 dimensional illustration of peak-to-peak intervals in individual rhythms of 6 genotypes

(a) Detrended circadian PER2::LUC rhythms in the neonatal SCN of different

genotypes. One representative rhythm is shown for each genotype. Arrow heads indicate peak of PER2::LUC rhythm, and the peak-to-peak intervals are indicated in different color with different period groups. (b) The distributions of peak-to-peak intervals in individual PER2::LUC rhythms are shown in 3 dimensional distribution maps for each genotype. Intervals (2h bin), frequency and individuality are indicated by X, Y and Z axis, respectively.
